# Association among obesity, overweight and autism spectrum disorder: a systematic review and meta-analysis

**DOI:** 10.1038/s41598-017-12003-4

**Published:** 2017-09-15

**Authors:** Zhen Zheng, Li Zhang, Shiping Li, Fengyan Zhao, Yan Wang, Lan Huang, Jinglan Huang, Rong Zou, Yi Qu, Dezhi Mu

**Affiliations:** 1 Department of Pediatrics, West China Second University Hospital, Sichuan University, Chengdu, 610041 China; 2 Key Laboratory of Birth Defects and Related Diseases of Women and Children, Ministry of Education, Sichuan University, Chengdu, 610041 China

## Abstract

Obesity, overweight and autism spectrum disorder (ASD) remain serious public health problems. Although lots of studies have recently explored the association among obesity, overweight and ASD, the findings are inconsistent. Thus, we conducted a meta-analysis of epidemiological studies to examine the association among obesity, overweight and ASD. PubMed, Embase, and the Cochrane Library were used for literature searches to identify eligible studies published in English before November 15, 2016. Relevant studies estimating the association among obesity, overweight and ASD were included. Fifteen studies encompassing 49,937,078 participants and 1,045,538 individuals with ASD were included in this study. A random effects model was chosen to synthesize the effect sizes of individual studies. The prevalence of obesity was significantly higher in individuals with ASD than in controls (OR = 1.84, 95% confidence interval [CI]: 1.37–2.48, *P* < 0.001). However, the prevalence of overweight in individuals with ASD was not significantly different from that in controls (OR = 1.07, 95% CI: 0.83–1.38, *P* = 0.62). Both sensitivity analysis and publication bias testing revealed that the findings were robust. The meta-analysis showed a significant association between obesity and ASD. However, no significant association was identified between overweight and ASD.

## Introduction

Obesity is a significant public health problem that affects 17% of children and 35% of adults in the United States^1^. Obesity can cause health problems including sleep-disordered breathing, orthopedic problems, hypertension, type 2 diabetes, and dyslipidemia^2–7^. Obesity is defined as an age-adjusted and gender-specific body mass index at or over the 95th percentile. Overweight is considered an age-adjusted and gender-specific body mass index between the 85th and 95th percentiles. Obesity or overweight harms psychosocial functions and is associated with significant societal and familial economic burdens^2,8^. Obese or overweight children are more susceptible to isolation and bullying than normal weight children^8^. Thus, obesity and overweight are becoming severe social issues and have a significant influence on quality of life and independent functioning.

Recently, scientists have reported that obesity and overweight are more likely to be related to psychiatric disorders in children, such as attention deficit/hyperactivity disorder and autism spectrum disorder (ASD)^9,10^. ASD is a complex disorder which manifests as difficulty in social interactions and communication, along with restrictive and repetitive behaviors. ASD is a complex and heterogeneous disorder that is due to the interaction effect between genetic vulnerability and environmental factors. The overall prevalence of ASD is 1 in 68 children^11^. Additionally, ASD is co-morbid with mental health disorders such as anxiety disorder and attention deficit/hyperactivity disorder but not with medical condition such as asthma and allergy^12–15^. Since ASD is a serious public health problem, scientists have attempted to identify biomarkers to achieve an earlier diagnosis and enable earlier treatment. We and other researchers have shown that biomarkers such as brain-derived neurotrophic factor and glutamate are sensitive markers for the early diagnosis of ASD and bipolar disorder^16–19^. However, specific biomarkers relevant only to ASD are still needed. Furthermore, specific and effective treatment for ASD in the clinic is scarce. ASD begins at an early age and lasts throughout the lifespan, which increases the economic burden on both family and society. Thus, ASD is also a significant global public health problem.

Individuals with ASD have several issues, including food selectivity, gastrointestinal symptoms, less physical activity, and medication use, which may result in abnormal anthropometric measurements^20–23^. Recently, some studies have focused on the association among obesity, overweight and ASD^9,24–26^. However, the findings are inconsistent. Some scientists have reported that BMI in individuals with ASD is not different from that in controls^9,24^, while other scientists have reported the prevalence of obesity and overweight is higher in individuals with ASD than in controls^25,26^. Therefore, we performed a meta-analysis to examine the association among obesity, overweight and ASD. We believe that a comprehensive evaluation of this critical public health problem may promote efforts to develop adequate interventional strategies in this population.

## Results

### Literature search

**Figure 1:**
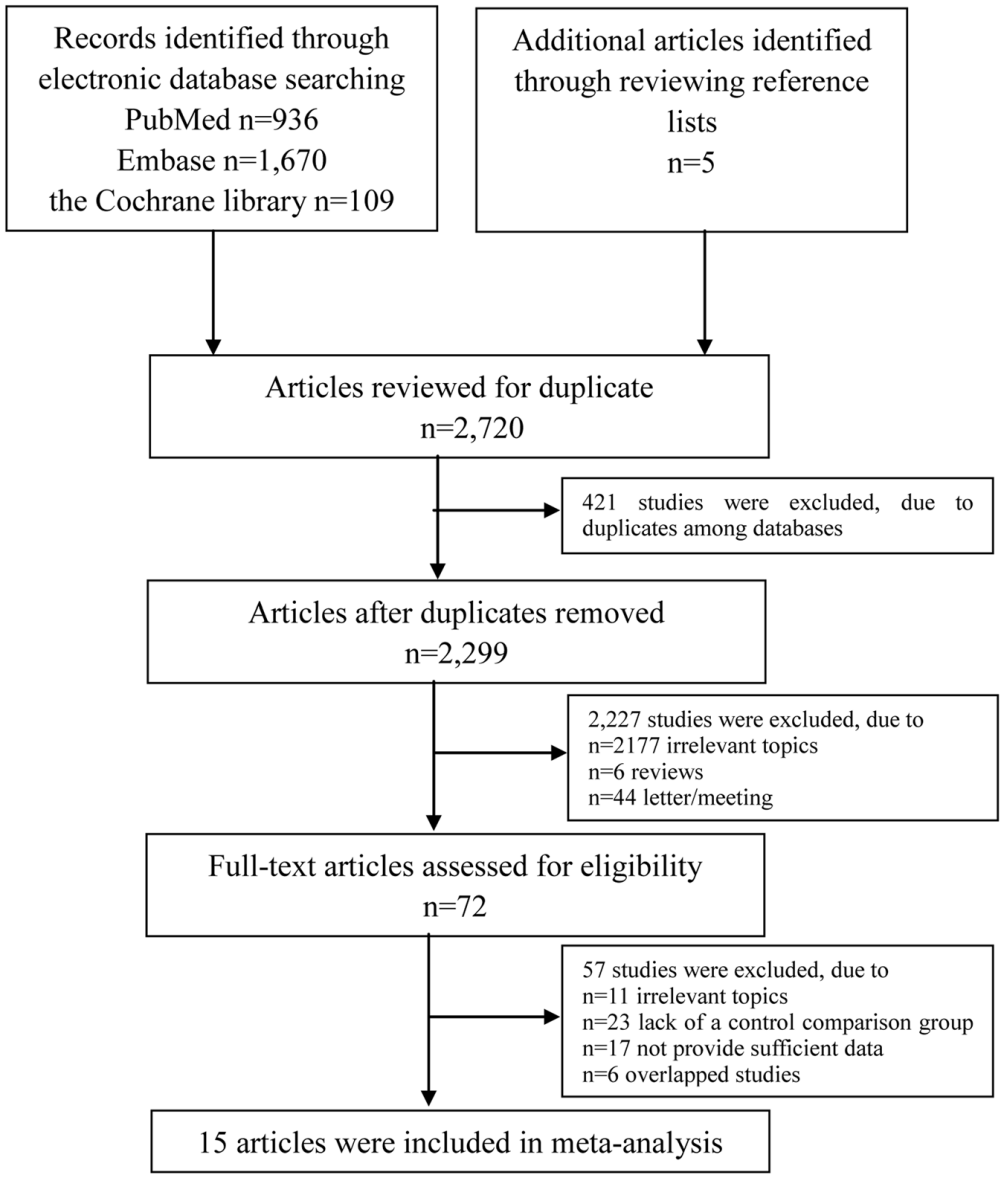
Flow diagram of the literature search. A total of 2,720 potential studies were initially identified in databases. 72 studies were identified after excluding 421 duplicate studies, 2,177 irrelevant studies, 6 reviews and 44 letters/meetings. Then, 57 studies were removed after a careful screening. Ultimately, 15 studies were adopted in this meta-analysis.

We initially identified a total of 2,720 potential articles, of which 936 were from PubMed, 1,670 were from Embase, 109 were from the Cochrane Library and 5 were from screening the references in the included articles. We identified 72 articles after excluding 421 duplicate articles, 2,177 irrelevant articles, 6 reviews and 44 letters/meetings. Then, those 72 articles were screened carefully. Eleven articles were excluded because their topics were irrelevant. Twenty-three articles were excluded because they lacked a control comparison group. Seventeen articles were excluded because they did not provide sufficient data and 6 articles were excluded because of overlap. Ultimately, 15 articles encompassing 49,937,078 participants and 1,045,538 individuals with ASD were included in this study. The flow diagram of the literature search is shown in Fig. 1.

### Study characteristics

The extracted data from the fifteen studies are shown in Table 1. Fifteen studies were published between 2010 and 2016^9,24–37^. Four studies were case-control^24,27,31,33^ and eleven studies were cross-section^9,25,26,28–30,32,34–37^. The studies encompassed a total of 49,937,078 participants and 1,045,538 individuals with ASD. Ten studies were carried out in the United States^24–27,29,30,34–37^, two in Brazil^9,31^, one in Turkey^32^, one in Spain^33^, and one in China^28^. The sample sizes ranged from 40^33^ to 986,352^30^ individuals with ASD and from 19^9^ to 48,598,466^30^ controls. Additionally, the mean ages of the samples ranged from 2 years old^25,27,34^ to 29.4 ± 12.1 years old^35^.

**Table 1:** Description of the studies in this meta-analysis.

First author, year	Country	Study design	Sample sizes ASD/control	Males(n) ASD/control	Mean age (year) ASD/control	Method of ascertaining ASD	Method of ascertaining BMI	Outcome variable	Adjusted confounders
Bandini^37^	USA	Cross-section	53/58	44/45	6.6 ± 2.1 6.7 ± 2.4	ADI-R	Objectively measured	Overweight	Age, gender
Barnhill^24^	USA	Case-control	86/57	79/47	66.19 ± 19.94 74.38 ± 22.3 (month)	SCQ, ADI-R, ADOS	Objectively measured	Overweight, obesity	Age, gender
Bicer^32^	Turkey	Cross-section	118/97	118/97	12–18 12–18	DSM-IV, DSM-V	Objectively measured	Overweight, obesity	Age, gender
Broder-Fingert^25^	USA	Cross-section	2976/3696	2359/1852	2–20 2–20	ICD-9	Extracted from medical record	Overweight, obesity	Age, gender, race/ethnicity, insurance type, autism-related medication use, select co-occurring conditions
Castro^31^	Brazil	Case-control	49/49	49/49	10.06 ± 3.82 10.02 ± 2.83	DSM-IV	Objectively measured	Overweight, obesity	Gender
Corvey^30^	USA	Cross-section	986352/48598466	816263/24540412	NR NR	Parental report	Parental report	Overweight, obesity	Age, gender, race, ethnicity, geographic location
Croen^35^	USA	Cross-section	1507/15070	1102/11020	29 ± 12.2 29.4 ± 12.1	ICD-9-CM	Extracted from medical record	Obesity	Age, gender, race/ethnicity
Healy^27^	USA	Cross-section	67/74	53/39	NR NR	Parental report	Objectively measured	Overweight, obesity	Age, gender
Hill^34^	USA	Cross-section	5053/8844	4270/4543	2–17 2–17	DSM-IV, ADOS	Objectively measured	Overweight, obesity	Age
Kummer^9^	Brazil	Cross-section	69/19	NR	8.4 ± 4.2 8.6 ± 2.9	DSM-V	Objectively measured	Overweight, obesity	NR
Liu^28^	China	Cross-section	154/73	141/67	5.21 ± 1.83 4.83 ± 0.84	DSM-V	Objectively measured	Overweight, obesity	Age, gender, the family structure, the minority percentage, parents’ education levels
Mari-Bauset^33^	Spain	Case-control	40/113	35/63	7.01 ± 1.01 8.34 ± 1.19	ADOS-G, ADI-R	Objectively measured	Overweight, obesity	NR
Phillips^36^	USA	Cross-section	93/8141	74/3826	12–17 12–17	Parental report	Parental report	Overweight, obesity	Age, gender, race/ethnicity, mother’s education, poverty-to-income ratio, birth weight
Rimmer^26^	USA	Cross-section	159/12973	NR	14.7 ± 1.9 NR	Parental report	Parental report	Overweight, obesity	Age, gender, race
Shedlock^27^	USA	Case-control	48762/243810	39010/195048	2–18 2–18	ICD-9-CM	Objectively measured	Obesity	Age, gender

Abbreviation: n = number, NR = not reported, BMI = body mass index.

For the method of ascertaining ASD, four studies used parental reports^26,29,30,36^, and the other studies based on their diagnoses on the Diagnostic and Statistical Manual (DSM-IV)^31,32,34^, DSM-V^9,28,32^, Social Communication Questionnaire (SCQ)^24^, International Classification of Diseases, 9th Revision (ICD-9)^25^, International Classification of Diseases, 9th Revision, Clinical Modification (ICD-9-CM)^27,35^, Autism Diagnostic Interview-Revised (ADI-R)^24,33,37^, Autism Diagnostic Observation Schedule (ADOS)^24,34^, and Autism Diagnostic Observation Schedule-Generic (ADOS-G)^33^. For the method of ascertaining BMI, three studies included parentally reported data^26,30,36^, ten studies objectively measured the data^9,24,27–29,31–34,37^, and two studies extracted the information from the participants’ medical records^25,35^.

### The prevalence of obesity and overweight in individuals with ASD

**Figure 2:**
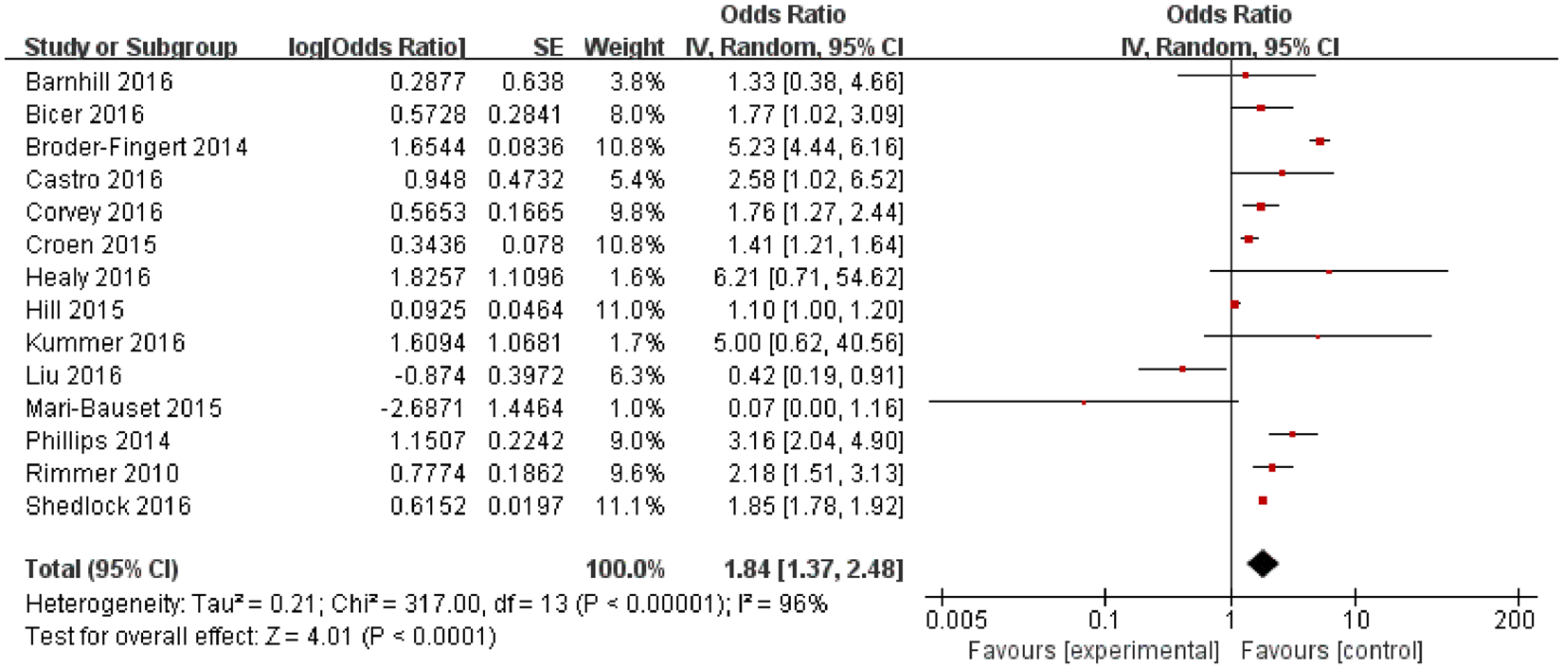
Forest plot of odds ratios for the association between obesity and ASD. Fourteen studies encompassing 1,045,485 individuals with ASD and 48,891,482 controls were included. The prevalence of obesity was higher in individuals with ASD than in controls. There was a significant association between obesity and ASD.

**Figure 3:**
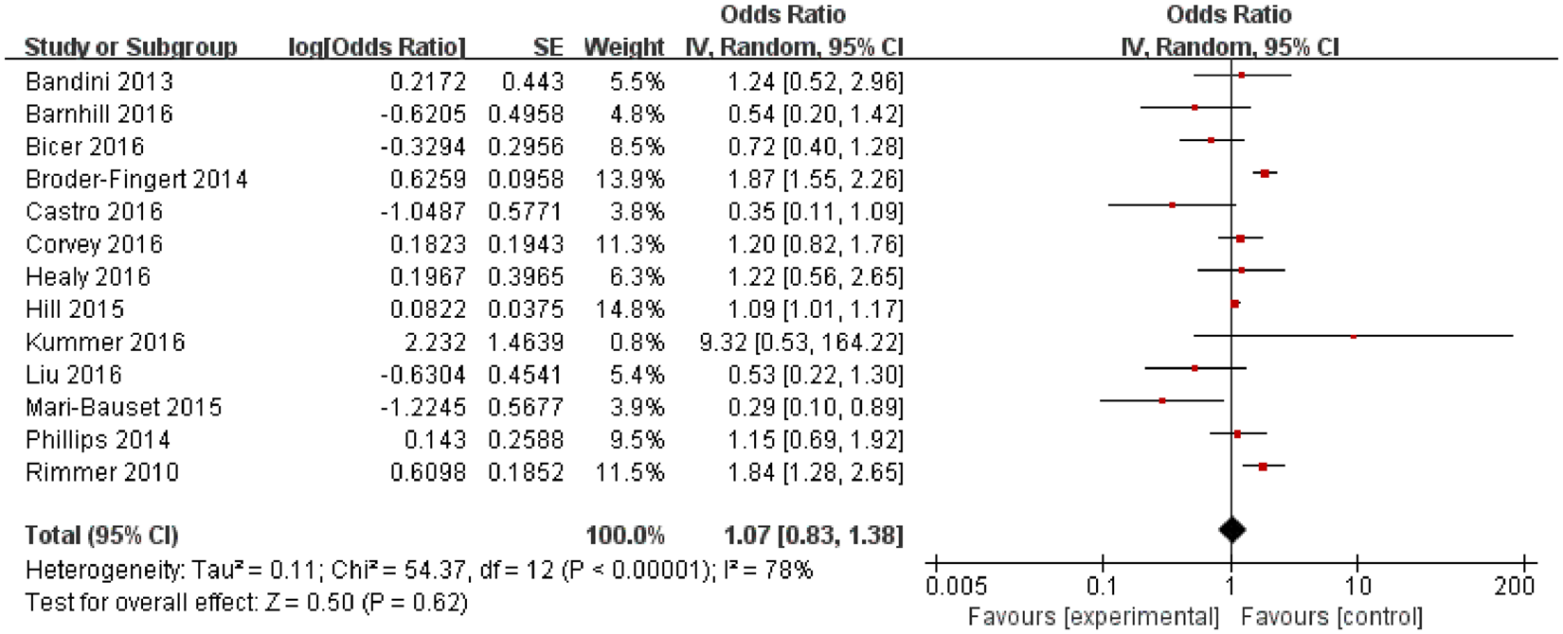
Forest plot of odds ratios for the association between overweight and ASD. Thirteen articles encompassing 995,269 individuals with ASD and 48,632,660 controls were included. The prevalence of overweight was not significantly different between individuals with ASD and controls. There was no significant association between overweight and ASD.

The prevalence of obesity was significantly higher in individuals with ASD than in controls (OR = 1.84, 95% confidence interval [CI]: 1.37–2.48, *P* < 0.001). Significant heterogeneity was found across studies (I^2^ = 96.0%, *P* < 0.001) (Fig. 2). However, the prevalence of overweight in individuals with ASD was not significantly different from that in controls (OR = 1.07, 95% CI: 0.83–1.38, *P* = 0.62). Significant heterogeneity was found across studies (I^2^ = 78.0%, *P* < 0.001) (Fig. 3).

### Quality evaluation

As presented in Table 2, eleven studies were considered high quality, while four studies were considered moderate quality. The mean score of all studies was 7.1.

**Table 2:** Quality evaluation by the Newcastle–Ottawa Scale.

First author, year	Study design	Selection	Comparability	Exposure/Outcome	Total scores
Bandini^37^	Cross-section	★★★★	★★	★★	8
Barnhill^24^	Case-control	★★	★★	★★	6
Bicer^32^	Cross-section	★★★★	★★	★★	8
Broder-Fingert^25^	Cross-section	★★★★	★★	★	7
Castro^31^	Case-control	★★★	★★	★★	7
Corvey^30^	Cross-section	★★★	★★	★	6
Croen^35^	Cross-section	★★★★	★★	★	7
Healy^27^	Cross-section	★★★	★★	★★	7
Hill^34^	Cross-section	★★★★	★★	★★	8
Kummer^9^	Cross-section	★★★★	★	★★	7
Liu^28^	Cross-section	★★★★	★★	★★	8
Mari-Bauset^33^	Case-control	★★★★	★	★★	7
Phillips^36^	Cross-section	★★★	★★	★	6
Rimmer^26^	Cross-section	★★★	★★	★	6
Shedlock^27^	Case-control	★★★★	★★	★★	8

### Publication bias

**Figure 4:**
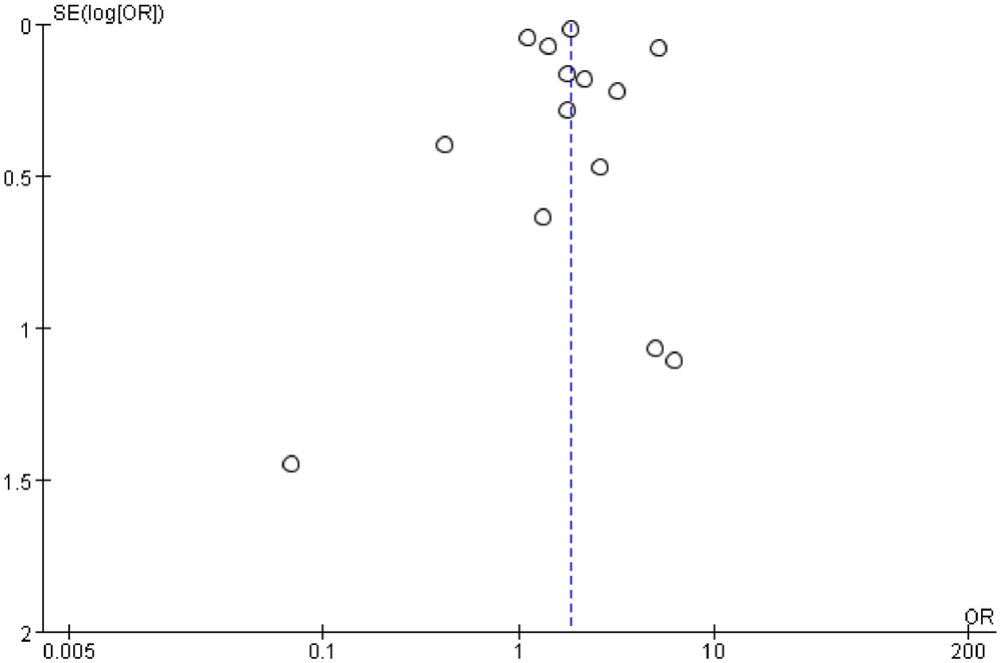
Funnel plot of the association between obesity and ASD. The pooled effect size is shown on the horizontal line. The expected 95% confidence interval for a given standard error is shown by the angled lines. The funnel plot showed potential publication bias.

**Figure 5:**
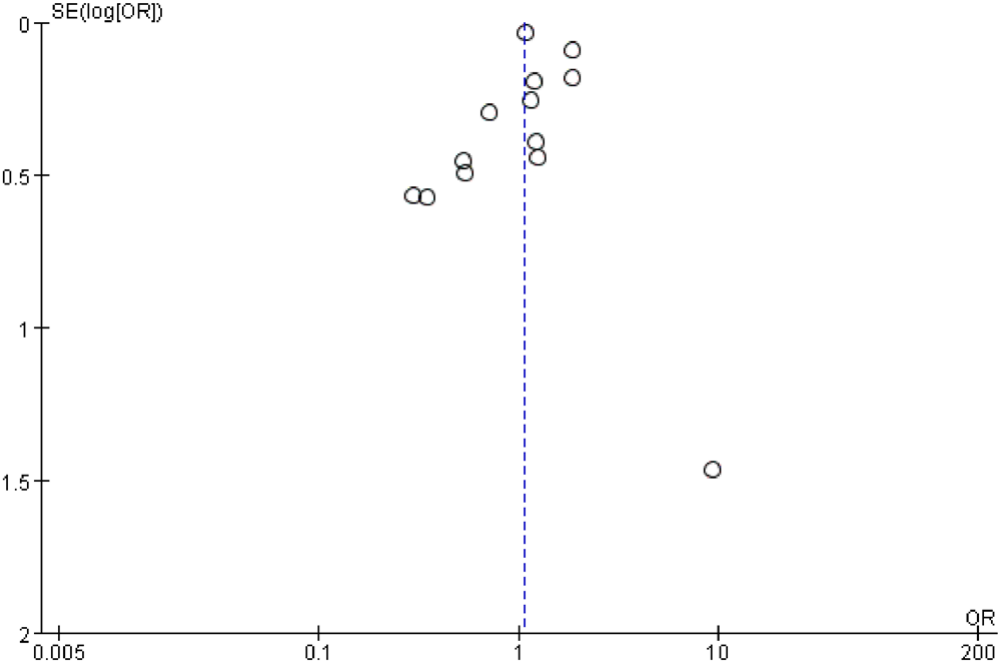
Funnel plot of the association between overweight and ASD. The pooled effect size is shown on the horizontal line. The expected 95% confidence interval for a given standard error is shown by the angled lines. The funnel plot showed potential publication bias.

The funnel plot showed slight asymmetry (Figs 4 and 5). However, Begg’s test (*P* = 0.913) and Egger’s test (*P* = 0.925) showed no significant publication bias among the studies examining the association between obesity and ASD. Similarly, Begg’s test (*P* = 0.246) and Egger’s test (*P* = 0.773) showed no significant publication bias among the studies examining the association between overweight and ASD.

### Subgroup analysis and sensitivity analysis

Subgroup analyses by study design, geographic locations, method of ascertaining of ASD, method of ascertaining BMI, and adjustment factors for age and gender were shown in Table 3.

**Table 3:** Summary of meta-analysis results on the association among obesity, overweight and ASD.

Variables	Obesity				Overweight			
	No. of comparisons	Meta-analysis OR (95%)	Heterogeneity I^2^ (*P*-value)	Test for subgroup difference I^2^ (*P-*value)	No. of comparisons	Meta-analysis OR (95%)	Heterogeneity I^2^ (*P*-value)	Test for subgroup difference I^2^ (*P*-value)
**All studies**	14	1.84 (1.37, 2.48)	96 (<0.001)		13	1.07 (0.83, 1.38)	78 (<0.001)	
**Study design**								
Case-control	4	1.63 (0.86, 3.09)	50 (0.11)	0 (0.96)	3	0.39 (0.21, 0.73)	0 (0.70)	91.2 (0.007)
Cross-section	10	1.92 (1.18, 3.14)	97 (<0.001)		10	1.24 (0.96, 1.60)	78 (<0.001)	
**Geographic locations**								
USA	9	2.08 (1.49, 2.91)	97 (<0.001)	18.8 (0.27)	8	1.30 (1.00, 1.70)	81 (<0.001)	85.5 (0.008)
non-USA	5	1.15 (0.42, 3.10)	77 (0.002)		5	0.55 (0.31, 0.98)	37 (0.17)	
**Method of ascertaining ASD**								
Parental report	4	2.28 (1.67, 3.11)	43 (0.15)	45.1 (0.18)	4	1.39 (1.09, 1.78)	14 (0.32)	75.1 (0.05)
Standard assessment	10	1.64 (1.13, 2.36)	97 (<0.001)		9	0.88 (0.61, 1.28)	83 (<0.001)	
**Method of ascertaining BMI**								
Objectively measured	9	1.38 (0.95, 2.00)	94 (<0.001)	57.2 (0.13)	9	0.79 (0.55, 1.13)	54 (0.02)	89.2 (0.002)
Others	5	2.45 (1.30, 4.65)	97 (<0.001)		4	1.56 (1.21,2.00)	55 (0.09)	
**Adjustment factors (Age and gender)**								
Yes	10	1.96 (1.39, 2.78)	95 (<0.001)	0 (0.5)	9	1.17 (0.87, 1.58)	68 (0.002)	0 (0.32)
No	4	1.39 (0.54, 3.59)	66 (0.03)		4	0.70 (0.26, 1.86)	73 (0.01)	

Among the studies examining the association between obesity and ASD, the tests for subgroup differences were not significant in the subgroup analyses by study design, geographic locations, method of ascertaining ASD, method of ascertaining BMI, and adjustment factors for age and gender.

Among the studies examining the association between overweight and ASD, the tests for subgroup differences showed significant different in the subgroup analyses by study design, geographic locations, and method of ascertaining BMI.

The sensitivity analysis was conducted by removing each study individually and comparing the results from the rest of studies with the overall result. The results showed that there was consistently a higher prevalence of obesity in individuals with ASD than in controls, while there was no significant different in the prevalence of overweight between individuals with ASD and controls. The omission of any single study did not change the overall result.

## Discussion

This meta-analysis included 15 studies with a total of 49,937,078 participants and 1,045,538 cases of ASD. The results showed that obesity but not overweight was associated with ASD.

In this study, we adopted a random-effects model to synthesize the effect sizes of individual studies because significant heterogeneity between studies existed. Sensitivity analysis revealed the findings were robust. In addition, both Begg’s and Egger’s tests indicated no significant publication bias existed.

An association between obesity and ASD was confirmed in this study. These findings are supported by previous studies. First, individuals with ASD often have eating problems, such as food selectivity and specialized dietary habits, which make healthy dietary interventions less effective^38,39^. Second, individuals with ASD spend less time on physical activities and more time on sedentary activities^29^. They also have difficulty in participating in standard obesity prevention initiatives due to behavioral problems. Third, individuals with ASD have more chances to be treated with various antipsychotic medications, which are known to be associated with weight gain^40,41^. Fourth, individuals with ASD also have comorbidities associated with obesity, such as sleep problems, gastrointestinal disorders, ADHD^13,23,42,43^. Finally, some individuals with ASD have been reported to have 16p11.2 or 11p14.1 microdeletions, which encompass genes related to obesity susceptibility^44,45^. These results indicated that obesity is related to ASD, which is consistent with our findings.

However, we found significant heterogeneity in this meta-analysis. Thus, we performed subgroup analyses to find the source of heterogeneity. But we could not find the source of heterogeneity among the studies examining the association between obesity and ASD (Table 3). Residual confounding factors such as different ages and genders across studies need to be considered. In addition, we found three factors that may explain the heterogeneity among the studies examining the association between overweight and ASD. First, the overall result indicated that there was no difference in the prevalence of overweight between individuals with ASD and controls in the USA samples, whereas the prevalence of overweight in individuals with ASD was lower than that in controls in the non-USA samples (Table 3). The result may be because of different genetic backgrounds and lifestyles. Second, different study designs (case-control or cross-section) were a cause of the heterogeneity (Table 3). Third, the different methods of ascertaining BMI (objectively measured, extracted from medical records or parentally reported) used in these studies contributed to heterogeneity (Table 3). The measures of ascertaining BMI in several studies biased the results because the data were collected from parental reports rather than objectively measured.

Although the heterogeneity is high, the analysis has certain obvious advantages. First, we are the first to conduct a meta-analysis to explore the association among obesity, overweight and ASD. Second, the meta-analysis included a huge amount of samples, making it more likely to draw a reasonable conclusion about obesity and overweight in ASD. Third, the sensitivity analysis showed that removing any study did not change the final results, suggesting that our findings were robust. Finally, publication bias was not found, which increased the reliability of the findings.

However, some limitations existed in this study. First, weight status was reported by parents in three studies. Therefore, the BMI may not be accurate in those studies. Future work should attempt to have the BMI measured objectively by trained clinicians. Second, there was significant heterogeneity across studies, which may reduce the conclusiveness of the results. Finally, adjustments were not made for confounding factors including dietary habits, physical activity participation, family history of obesity and secondary conditions. ASD is a heterogeneous disorder. Secondary conditions, including physical status, mental health status, developmental delay, intellectual and learning disabilities, attention deficit/hyperactivity disorder, IQ and drugs use might influence the association between obesity and ASD^30^. Therefore, future research should systematically adjust for a broad set of possible confounding factors.

In conclusion, this meta-analysis showed a significant association between obesity and ASD. However, no significant association was identified between overweight and ASD. Further prospective studies with more accurate measures of weight status and better control of confounding factors are warranted.

## Materials and Methods

### Strategy of literature search

We performed a literature search of PubMed, Embase and the Cochrane Library for the potential articles. The search was restricted to articles published before November 15, 2016. The search terms were as follows: [“ASD” OR “autism spectrum disorder” OR “autistic disorder” OR “autism” OR “Asperger syndrome” OR “pervasive developmental disorder”] and [“obesity” OR “obese” OR “overweight” OR “adipose” OR “adiposity” OR “body weight” OR “body mass index” OR “BMI”]. Furthermore, we searched the references of the related articles to attain other potential studies.

Only studies published in English were considered. In addition, we only considered studies performed on human. We reviewed the titles and abstracts to exclude the irrelevant studies. Then, we read the text carefully based on the inclusion criteria. If two authors reached inconsistent conclusions about inclusion/exclusion, a third author would be asked to make a decision.

### Study selection criteria

Any study that reached all the following criteria was included: (1) assessing the association among obesity, overweight and ASD; (2) reporting the raw data or OR with 95% CI; and (3) case-control, cohort or cross-sectional designs.

The following studies were excluded: (1) reviews, non-human studies, case reports, case-only studies, or meetings/letters; and (2) overlapping data.

### Data extraction

The data were extracted from the studies as follows: first author’s surname, year of publication, country, study design, numbers of cases and controls, number of males, age, methods of ascertaining ASD and BMI, outcome variable and adjusted confounders. If multiple studies contained overlapping data from the same population, the one with the largest sample size was adopted.

### Quality evaluation

We conducted Newcastle-Ottawa Scale (NOS) to evaluate the quality of the studies. The NOS evaluated the following aspects: selection of participants, comparability, exposure of participants, and outcome. The score ranged from 0 to 9. Scores of 7–9 indicate high quality, scores of 4–6 indicate medium quality and scores of 0–3 indicate low quality. When raters disagreed, the inconsistencies were settled by discussion.

### Statistical analysis

We combined the effect sizes by using ORs to assess the association among obesity, overweight and ASD. We calculated the ORs by using a fixed-effects model or a random-effects model. If the heterogeneity was low, the fixed-effects model was used. If the heterogeneity was high, the random-effects model was chosen. I^2^ and the Q statistic were adopted to evaluate the heterogeneity. The heterogeneity was divided into the following levels according toI^2^ value: low (I^2^: 25%-50%), moderate (I^2^: 50–75%) and high (I^2^: >75%). The Q statistic was viewed significant if *P* < 0.1.

The funnel plot was visually inspected to evaluate publication bias. In addition, both Begg’s and Egger’s tests were conducted to evaluate publication bias. Publication bias was viewed significant if *P* < 0.05. Subgroup analyses were conducted to identify the sources of heterogeneity based on study design (case-control or cross-section), geographic locations (USA or non-USA), methods of ascertaining ASD (parental report or standard assessment), methods of ascertaining BMI (objective measurement or other methods), and adjustment factors for age and gender (yes or no).

Sensitivity analysis was conducted by individually omitting one study from statistical analysis. Then, the ORs and 95% CIs of the remaining studies were compared to the overall result. Statistical analyses were conducted with Stata 12.0 (Stata Corp, College Station, Texas, USA).
